# Wharton's jelly mesenchymal stem cell-derived conditioned media inhibits colon cancer cells via activating AMPK/mTOR-mediated autophagy

**DOI:** 10.22099/mbrc.2025.52891.2133

**Published:** 2026

**Authors:** Dian Dayer, Zahra Akbari-Jonoush, Roya Mahdavi, Afshin Amari, Amirhesam Keshavarz-Zarjani, Layasadat Khorsandi

**Affiliations:** 1 Cellular and Molecular Research Center, Medical Basic Sciences Research Institute, Ahvaz Jundishapur University of Medical Sciences, Ahvaz, Iran; 2 Department of Immunology, School of Medicine Ahvaz Jundishapur University of Medical Sciences, Ahvaz, Iran; 3 Cellular and Molecular Research Center, Birjand University of Medical Sciences, Birjand, Iran; 4 Department of Anatomical Sciences, Faculty of Medicine, Ahvaz Jundishapur University of Medical Sciences, Ahvaz, Iran

**Keywords:** Colon Cancer, AMPK, Stem Cell, Autophagy

## Abstract

Prior studies have shown that conditioned media derived from Wharton's jelly mesenchymal stem cells (WJ-CM) have anti-cancer properties. This research investigated the impact of WJ-CM on HT-29 colorectal adenocarcinoma cells by examining autophagy biomarkers and the AMPK/mTOR pathway. The HT-29 cells were subjected to WJ-CM and an AMPK activator (AICAR). Autophagy and levels of AMPK and mTOR proteins were investigated. WJ-CM increased the expression of phosphorylated AMPK while reducing the level of phosphorylated mTOR in HT-29 cells. WJ-CM treatment elevated the LC3B/LC3A ratio and ATG7, ATG5, and Beclin-1 expression. However, there was a parallel drop in p62 expression, which indicates autophagy induction. AICAR increased the influence of WJ-CM on viability, as well as the levels of biomarkers associated with autophagy, phosphorylated  AMPK, and phosphorylated  mTOR in the HT-29 cells. WJ-CM inhibits colorectal cancer cell growth via activating AMPK/mTOR-mediated autophagy.

## INTRODUCTION

Colorectal cancer (CRC) is a common malignancy that frequently presents with a poor prognosis. The current therapeutic methods for CRC are accompanied by several side effects [1]. Many researchers have investigated the influence of mesenchymal stem cells (MSCs) on various types of malignancies. However, the results are varied and controversial due to differences in mesenchymal cells, tumor type, and animal models [2-9]. MSCs secrete various cytokines, chemokines, and growth factors into their culture medium [10]. These biomaterials can promote or suppress tumor cell growth [11, 12].

Wharton's jelly (WJ) from the umbilical cord is suitable for obtaining stem cells using non-invasive techniques, which can be readily cultured on a large scale without raising any ethical concerns. WJMSC prevents the progression of several types of malignant cells, such as cervical cancer, leukemia, and CRC cells [13-15]. Cancer cells under oxidative stress, hypoxia, and nutritional deficiency activate AMPK signaling, which inhibits the mTOR, thereby promoting autophagy [16]. mTOR modulates cell viability, autophagy, and apoptosis of cancerous cells [17]. AMPK and mTOR also have a signaling relationship, and the AMPK-mTOR axis regulates autophagy [18]. 

When AMPK is phosphorylated, tumor progression is prevented by suppressing the mTOR pathway and inducing autophagy in various malignant cells. On the other hand, the inactivation of AMPK encourages tumor development [19]. There has been no report indicating whether the AMPK/mTOR axis is related to the influence of WJMSC on CRC cells. This study investigated the impact of conditioned media (CM) containing substances secreted by stem cells derived from WJ (WJ-CM) on AMPK/mTOR-dependent autophagy in a human CRC cell line (HT-29).

## MATERIALS AND METHODS

### Experimental design:

WJMSCs (PCS-500-010™) and the HT-29 cells (C466) were from the GEN IRAN and Pasteur Institutes (Iran). After characterization, the WJMSCs were grown in HG-DMEM (Sigma, USA) enriched with routine Pen/Strep (Sigma, USA) and fetal bovine serum (FBS) (Gibco, Germany). When the WJMSCs reached 75% confluence (approximately 3 days), CM was collected and stored at -70°C. The cells were maintained in RPMI medium (Sigma, USA) enriched with Pen/Strep and FBS. These cells were incubated in an environment of 5% CO_2_, 95% humidity, and a temperature of 37°C for 24 h. Three groups were used in this experiment: Control: only with the medium for 24 h; WJ-CM: WJ-CM for 24 h (see Table S1); WJ-CM + AICAR (an AMPK activator): 100 μM AICAR+WJ-CM for 24 h.

### MTT Assay:

HT-29 cells were grown on 96-well plates, with 10,000 cells per well and subjected to three different treatments: medium only (control), WJ-CM, and WJ-CM combined with AICAR, for 24 h. After exposure, MTT was incubated at 37°C for 4 h at 37°C. The supernatants were replaced with Dimethyl sulfoxide (DMSO), and the optical densities at 570 nm were recorded.

### Colony formation:

 HT-29 cells (500 cells/well) were cultivated and received either medium alone or media enriched with WJ-CM, with or without AICAR. The cells were maintained at 37˚C in a 5% CO_2_ environment for two weeks, until the untreated HT-29 cells (control group) formed colonies. The colonies containing at least 50 cells were counted.

### Gene Expression Analysis via qRT-PCR:

RNA extract was prepared using Parstous RNA extraction kit (A101231, Iran) and subsequently converted into cDNA by Parstous cDNA synthesis kit (A101162, Iran). The prepared cDNA was mixed with Master Mix and primers (Table S2).  A 45-cycle protocol was employed for PCR amplification utilizing the AB One-Step apparatus from Germany. Gene expression was evaluated by the 2^-ΔΔCT^ formula, using GAPDH as a calibrator.

### Western Blot Analysis:

The treated cells were homogenized, and their proteins were extracted using the Bradford kit. The extracted proteins were subsequently transferred to a polyvinylidene difluoride membrane. The membranes underwent a blocking procedure and were exposed to primary antibodies diluted in Tris buffer containing 5% BSA (Bovine serum albumin) for 18 h at 4°C. The membranes were then exposed to the secondary antibody for 2 h and subsequently developed using an ECL kit (Sigma).

### Statistical Analysis:

Data were analyzed using the one-way analysis of variance, accompanied by Tukey's or Kruskal-Wallis tests (employing SPSS 21.0 software). *p*-value < 0.05 was considered statistically significant.

## RESULTS

**Figure 1:**
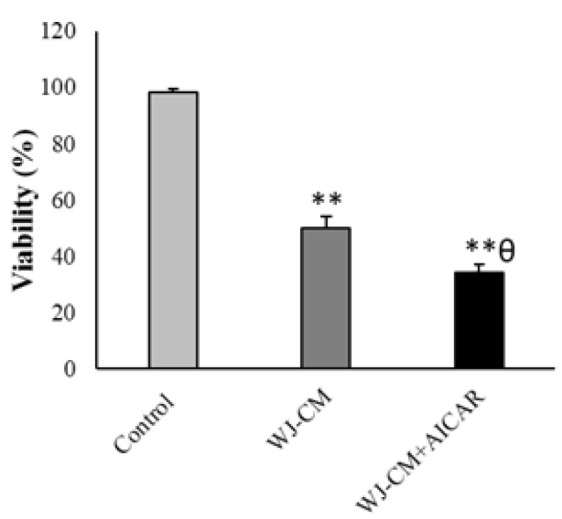
Viability percentage of the HT-29 cells (mean ± SD; n=6). **p<0.01, ^θ^*p*<0.05, *and ^θ^show comparison to the control and WJ-CM groups.

**Figure 2:**
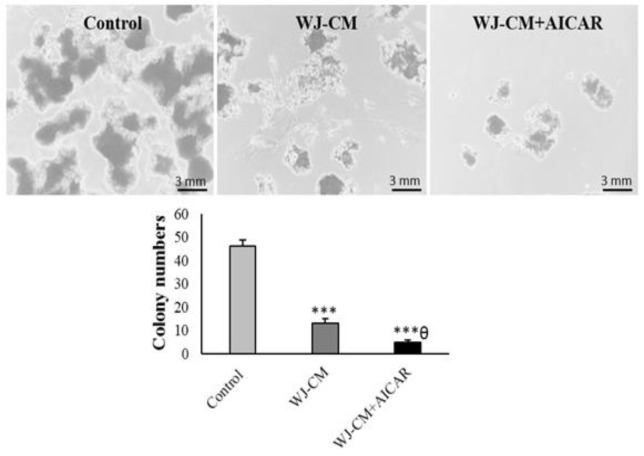
Colony number of the HT-29 cells (mean ± SD; n=3). ***p<0.001,^ θ^ *p*<0.05; *and ^θ^display comparison to the control and WJ-CM treatment.

The MTT assay indicated that WJ-CM diminished the viability of cancer cells. The cells treated with WJ-CM + AICAR showed an even greater reduction in viability than the cells treated with WJ-CM alone (Fig. 1). Additionally, WJ-CM significantly diminished the colony generation of HT-29 cells . The WJ-CM + AICAR-treated group presented significantly reduced colony numbers in comparison to the group that received WJ-CM treatment alone (Fig. 2).

**Figure 3:**
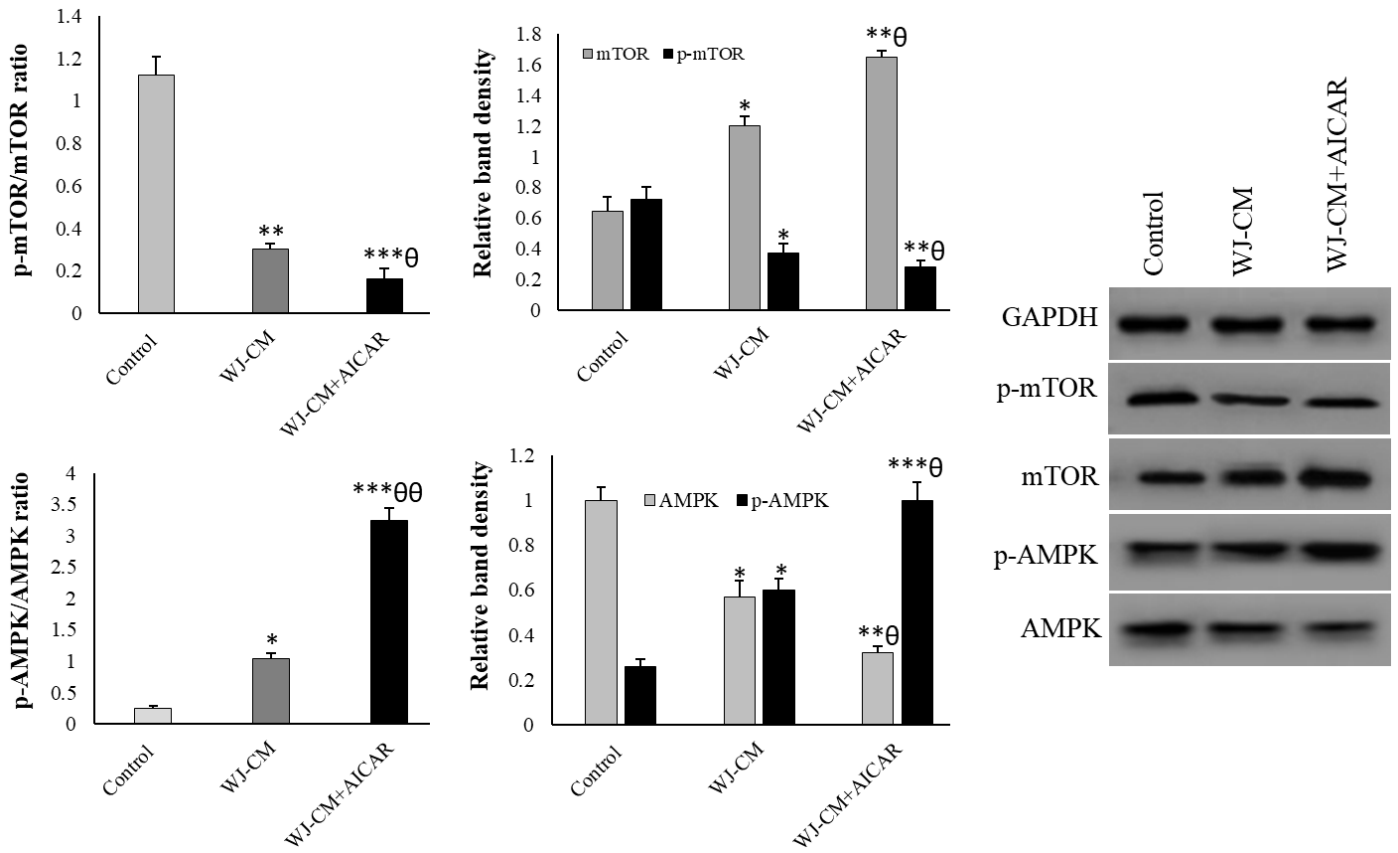
Western blotting results of relative expression of AMPK and mTOR proteins (mean ± SD; n=3). *p<0.05, **p<0.01, ***p<0.001,^ θ^*p*<0.05,^ θθ^*p*<0.01; *and ^θ^display comparison to the control and WJ-CM treatment.

WJ-CM significantly increased the p-AMPK (phosphorylated AMPK)/AMPK ratio in HT-29 cells. WJ-CM, on the other hand, reduced the HT-29 cells' p-mTOR (phosphorylated mTOR) to mTOR protein ratio. WJ-CM and AICAR integration increased p-AMPK protein levels while concurrently decreasing p-mTOR levels in comparison to the WJ-CM group (Fig. 3).

**Figure 4:**
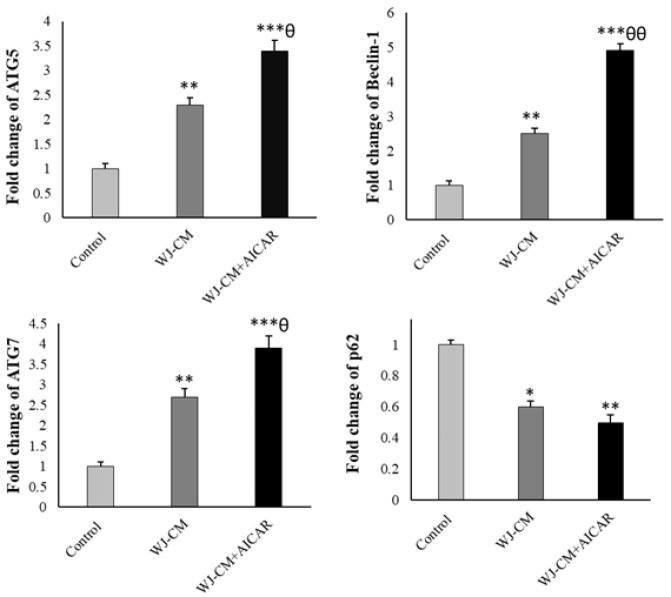
mRNA expression of autophagy-related genes (mean ± SD; n=3). *p<0.05, **p<0.01, ***p<0.001,^ θ^*p*<0.05,^ θθ^*p*<0.01; *and ^θ^display comparison to the control and WJ-CM treatment.

**Figure 5:**
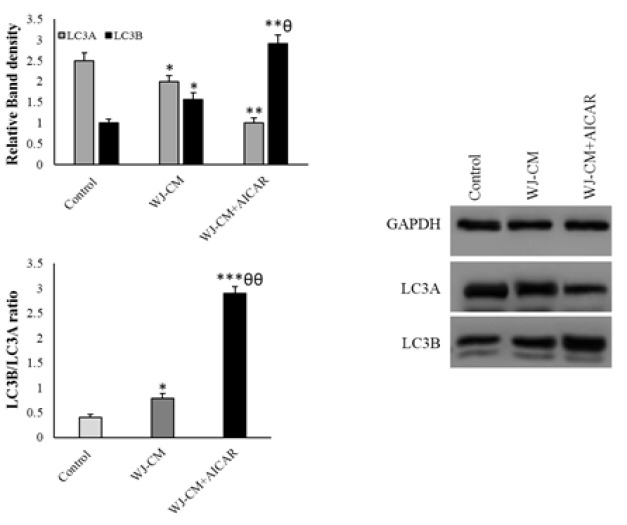
Autophagy assessments in the HT-29 cells (mean ± SD; n=6). *p<0.05, **p<0.01, ***p<0.001, ^θ^*p*<0.05,^ θθ^*p*<0.01, *and ^θ^display comparison to the control and WJ-CM groups.

The administration of WJ-CM increased the expression of the Beclin-1, *ATG5*, and *ATG7* genes, and decreased the expression of the *p62* gene. Results from Western blotting indicated that WJ-CM significantly enhanced the LC3B/LC3A ratio. AICAR plus WJ-CM significantly enhanced the *ATG5*, *ATG7*, Beclin-1, and *LC3B*, and decreased *p62* expression (Fig. 4 and 5).

## DISCUSSION

WJ-CM suppressed cell proliferation, induced autophagy, elevated p-AMPK levels, and reduced p-mTOR levels in HT-29 cells. Consistent with our findings, Wan et al. (2023) observed that CM of umbilical cord MSCs suppressed proliferation and survival of a granulosa tumor cell line [19]. Widowati et al. found that WJ-CM decreased the survival rate and induced apoptosis in MCF7 and T47D breast cancer cells [20].

The reduced viability of the WJ-CM may be caused by apoptosis in the HT-29 cells. Although apoptosis was not assessed in this study, previous studies have shown the apoptotic impacts of WJMSCs-derived secretome or MSC-derived CM on CRC cells [15, 21]. CM isolated from MSCs of umbilical cord and adipose tissue encourages apoptosis in the human glioma cells [22].

In our study, decreased viability was aligned with decreased p-mTOR and increased p-AMPK proteins in the WJ-CM-exposed cells. Increasing p-mTOR leads to the overexpression of proteins that promote CRC, and targeting mTOR can induce cancer cell death [23]. The mTOR signaling pathways facilitate the proliferation of gastric cancer cells [24]. MSCs derived from the umbilical cord prevented growth and diminished the level of mTOR in melanoma cells [6]. AMPK directly enhances autophagy by phosphorylating proteins associated with autophagy [25]. AMPK activation affects autophagy, apoptosis, and the cell cycle checkpoints [26].

This research utilized AICAR to confirm the contribution of AMPK in the WJ-CM-triggered HT-29 cells. The results demonstrate that AICAR, when used in conjunction with WJ-CM, significantly prevented the growth of HT-29 cells. WJ-CM, with or without AICAR, reduced the p-mTOR expression and enhanced the p-AMPK level in the HT-29 cells. Therefore, WJ-CM has the potential to function as an activator of AMPK and autophagy in the CRC cells. Safari and colleagues have reported that the secretome derived from amniotic MSCs promotes autophagy-dependent cell death in pancreatic cancerous cells [27]. To determine autophagy, the expression of autophagy-related biomarkers was analyzed. WJ-CM enhanced the LC3B to LC3A ratio and boosted ATG7, ATG5, and Beclin-1 expression while concurrently reducing the expression of p62, thereby inducing autophagy. The results suggest that WJ-CM is capable of effectively promoting autophagy in HT-29 cells. WJ-CM may trigger autophagy in CRC cells by modifying the AMPK/mTOR axis. Other research has also reported that AMPK/mTOR-dependent autophagy inhibits CRC growth [28-31]. Kong et al. have reported that Larotrectinib markedly inhibits CRC cell growth via activating AMPK/mTOR-mediated autophagy [32].

In conclusion, the higher p-AMPK and lower p-mTOR protein levels suggest that WJ-CM stimulates autophagy through AMPK/mTOR axis modulation. WJ-CM may be a therapeutic option for managing CRC.

### Acknowledgements:

This article is supported by the research council of Ahvaz Jundishapur University of Medical Sciences with grant number: CMRC-0242

### Conflict of Interest:

We declare that there are no conflicts of interests associated to this manuscript. 

### Ethics approval:

This project is approved by the Ethics committee of Ahvaz Jundishapur University of Medical Sciences (approved number: IR.AJUMS.REC.1402.693).

### Authors’ Contribution:

l.K. designed the study and supervised the experiments, D.D., R.M., and ZAJ contributed in performing experiments and collecting data. A.A. and A.K.Z. made contributions in interpreting data. All these authors have substantial contributions to the final manuscript and approved this submission.
