# Supplementary material for: Wharton's jelly mesenchymal stem cell-derived conditioned media inhibits colon cancer cells via activating AMPK/mTOR-mediated autophagy
Source: Mol Biol Res Commun. 2026;15(1):31–8. doi: 10.22099/mbrc.2025.52891.2133 (PMC12673627; doi:10.22099/mbrc.2025.52891.2133)
Supplement: Supplementary file 1 — Table S1-S2 [file mbrc-15-31-s001.pdf]

Molecular Biology Research Communications 2026;15(1):31-38 DOI:10.22099/mbrc.2025.52891.2133 MBRC

Original Article Open Access

Wharton's jelly mesenchymal stem cell-derived conditioned media inhibits colon cancer cells via activating AMPK/mTOR-mediated autophagy

Dian Dayer[1], Zahra Akbari Jonoush[1, 2], Roya Mahdavi[2, 3], Afshin Amari[1,2], Amirhesam Keshavarz-Zarjani[1, 4], Layasadat Khorsandi[1,4,*]

1) Cellular and Molecular Research Center, Medical Basic Sciences Research Institute, Ahvaz Jundishapur University of Medical Sciences, Ahvaz, Iran
2) Department of Immunology, School of Medicine Ahvaz Jundishapur University of Medical Sciences, Ahvaz, Iran
3) Cellular and Molecular Research Center, Birjand University of Medical Sciences, Birjand, Iran
4) Department of Anatomical Sciences, Faculty of Medicine, Ahvaz Jundishapur University of Medical Sciences, Ahvaz, Iran

Table S1: The Effect of WJ-CM on the viability of HT-29 cells at different time.

Treatment	Viability (%)
**12 hours**	87.4 ± 2.1
**24 hours**	49.8 ± 3.4*
**48 hours**	33.9 ± 3.1**

Values are expressed as mean ± SD (n=5). * $p < 0.05$, ** $p < 0.05$; * indicate comparison to 12 hours.

Table S2: Primer sequences.

Genes	Forward	Reverse
**p62**	GCTCAGGAGGAGACGATGAC	AGAAACCCATGGACAGCATC
**Beclin-1**	CGGTTTTTCTGGGACAACAA	AAAAACGTGTCTCGCCTTTC
**ATG5**	CCAGAAAAAGACCTTCTGCACT	CAATCCCATCCAGAGTTGCT
**ATG7**	ACCTTGGGTTGCAATGTAGC	CTTACCACCCCCTAGGCAAT
**GAPDH**	GCTGGACATTGGACTTCCTC	ACCACTGTGACCTGCTCCA
